# Anti-obesity effects of *Spirulina platensis* protein hydrolysate by modulating brain-liver axis in high-fat diet fed mice

**DOI:** 10.1371/journal.pone.0218543

**Published:** 2019-06-20

**Authors:** Bingli Zhao, Yujiao Cui, Xiaodan Fan, Ping Qi, Chunchen Liu, Xuesong Zhou, Xuewu Zhang

**Affiliations:** 1 College of Food Science and Engineering, South China University of Technology, Guangzhou, China; 2 Guangzhou Institute for Food and Drug Control, Guangzhou, China; 3 Zhongci Health Care Products Technology Development Co. Ltd, Guangzhou, China; 4 Guangzhou Honsea Industry Co. Ltd, Guangzhou, China; University of Southern Queensland, AUSTRALIA

## Abstract

*Spirulina platensis* is a blue-green algae with potential anti-obesity effects. In this study, the anti-obesity effects of whole *Spirulina platensis* (WSP), *Spirulina platensis* protein (SPP) and *Spirulina platensis* protein hydrolysate (SPPH) were compared in high-fat diet fed mice, and the potential acting mechanism of SPPH was also investigated. Totally, SPPH exhibited good anti-obesity effects (reducing 39.8%±9.7% of body weight), lowering 23.8%±1.6% of serum glucose, decreasing 20.8%±1.4% of total cholesterol, while positive drug Simvastatin had the corresponding values: 8.3%±4.6%, 24.8%±1.9% and -2.1%±0.2%, respectively. Subsequently, PCR array was used to conduct gene expression analysis in brain and liver tissues of SPPH-treated mice, which displayed distinctly different expression pattern. The most markedly changed genes included: Acadm (-34.7 fold), Gcg (2.5 fold), Adra2b (2 fold) and Ghsr (2 fold) in brain; Retn (39 fold), Fabp4 (15.5 fold), Ppard (6 fold) and Slc27a1 (5.4 fold) in liver. Further network analysis demonstrated that the significantly expressed genes in brain and liver tissues were mapped into an interacting network, suggesting a modulatory effect on brain-liver axis, major pathways were involved in the axis: PPAR, adipocytokine, AMPK, non-alcoholic fatty liver disease and MAPK. This study showed that *Spirulina platensis* protein hydrolysate possessed anti-obesity effect in mice.

## Introduction

The increasing consumption of high-fat diets and “fast food” leads to more and more obesity, which has become a major public health concern in the world. Obesity was considered to be association with chronic health disorders such as hyperlipidemia, hypertension, cardiovascular diseases, non-alcoholic fatty liver, insulin resistance and osteoarthritis [1]. Mankind has been fighting against obesity, however, the adverse side effects and rebound weight gain are major challenge for conventional drug therapies of obesity. There are continuing needs to develop safe and effective remedies to treat obesity.

Many nutritional intervention studies have showed that food-derived proteins or hydrolysates can reduce body weight and fat mass, and lower plasma cholesterol and triglycerides. Such as, proteins of white lupin seed were reported to reduce cholesterolemia in rats [2]; germinated soy protein hydrolysates had inhibitory effect on lipid storage and stimulating activity on lipolysis in 3T3-L1 cells [3]; yeast hydrolysate suppressed body fat accumulation by attenuating fatty acid synthesis in high-fat-diet-induced obese mice [4]; dietary fish protein hydrolysates were found to influence serum lipids and postprandial glucose regulation in obese Zucker fa/fa rats [5]; enzymatic hydrolysate from velvet antler significantly reduced the body weight gain, lowered the serum glucose and triglyceride levels in high-fat diet-fed mice [6]; yellow catfish protein hydrolysate displayed anti-obesity effects in high-fat diet fed mice, compared with those of mice treated with simvastatin [7]. The potential anti-obesity mechanisms include appetite regulation, lipid digestion/absorption intervention, fatty acid oxidation, regulation of lipogenesis and lipolysis [1]. At molecular level, PPAR family, primarily expressed in adipose tissue, liver and skeletal muscle, plays essential role in lipid metabolism [8]. By inactivation of acetyl-CoA carboxylase and carnitine palmitolytransferase-1, AMP-activated protein kinase (AMPK) plays a significant role in lipogenesis and fatty acid oxidation [9].

Recently, algae are considered to be a good source for anti-obesity agents [10]. Jung et al. [11] reported that the edible brown alga Ecklonia stolonifera and its constituent fucosterol decreased lipid accumulation in 3T3-L1 pre-adipocytes by inhibiting the expression of the adipocyte marker proteins peroxisome proliferator-activated receptor γ (PPARγ) and CCAAT/enhancer-binding protein α (C/EBPα). Choi et al. [12] indicated that a major phlorotannin dieckol from Ecklonia cava decreased body weight gain (38%) in high-fat diet-fed mice by activating AMP-activated protein kinase (AMPK) signaling. Kang et al. [13] reported that oral administration of Plocamium telfairiae extract significantly reduced the body weight, fatty liver, amount of white adipose tissue, and levels of triglyceride and glucose in obese mice fed a high-fat diet. Indole derivatives isolated from brown alga Sargassum thunbergii were shown to inhibit adipogenesis through AMPK activation in 3T3-L1 Preadipocytes [14]. In another study, they also showed that gelidium amansii extract ameliorates obesity by down-regulating adipogenic transcription factors in diet-induced obese mice [15]. Saringosterol isolated from Sargassum muticum exhibited anti-obesity effect by inhibiting the mRNA and protein expression of peroxisome proliferator-activated receptor γ and CCAAT enhancer-binding protein α in 3T3-L1 cells [16].

*Spirulina platensis* is a blue-green algae that can be consumed by humans and other animals. Park and Lee [17] demonstrated that Spirulina supplementation significantly decreased plasma concentration of total cholesterol and LDL-cholesterol in non-obese Korean elderly, but these effects were not observed in the obese group. Zeinalian et al. [18] reported that Spirulina supplementation in sixty four obese individuals aged 20–50 years, at a dose of 1 g/d for 12 weeks, decreased body weight, appetite and serum total cholesterol, and increased serum high density lipoprotein-cholesterol concentrations (HDL-c), but did not significantly affect low density lipoprotein-cholesterol and triglycerides. In our previous study, *Spirulina platensis*-derived hydrolysates were characterized and peptides were identified, which exhibited good inhibition on lipase and 3T3-L1 preadipocytes [19]. Here, it is hypothesized that *Spirulina platensis*-derived hydrolysate also possess the in vivo anti-obesity effect, and its acting mechanism may be related to gene expression changes in brain and liver. The objective of this study is to investigate anti-obesity effects of whole *Spirulina platensis* (WSP), *Spirulina platensis* protein (SPP) and *Spirulina platensis* protein hydrolysate (SPPH) in HFD-fed mice, and the potential acting mechanism of SPPH were also explored.

## Materials and methods

### Chemicals

Pepsin (1:3000 U/mg) was purchased from Qiyun Biotech Co. Ltd, Guangzhou, China. Bio-Rad Protein Assay Kit was from Bio-Rad Laboratories Inc., USA. Simvastatin was from Yuanye Biotech Co. Ltd, Shanghai, China. Glucose assay kit #10009582 was from Cayman Chemicals, USA. Total cholesterol (TC), triacylglycerol (TG), high-density lipoprotein (HDL)-cholesterol and low-density lipoprotein (LDL)-cholesterol detection kits were purchased from Nanjing Jiancheng Bioengineering Institute (Nanjing, China). All chemical reagents were at analytical grade.

### Sample preparations and assays

Whole *Spirulina platensis* (WSP): forty grams of *Spirulina platensis* powder (Ordos Biotech Co. Ltd, China) were suspended in 800 mL of pure water to obtain WSP.

*Spirulina platensis* protein (SPP): WSP was frozen at -20 ^o^C for 5 h and thawed out at room temperature, repeated for 4 times. Then, ultrasonication (450 W for 30 min, 9 s interval every 6 s) was performed in ice bath, the mixture was centrifuged at 8694×g (4°C, 45 min), and the supernatant was collected as SPP [20].

*Spirulina platensis* protein hydrolysate (SPPH): four grams of SPP were dissolved in pure water to obtain 3% (v/v) of protein solution. Hydrolysis was performed with pepsin under the conditions of pH 2, temperature 37 ^o^C and enzyme to substrate ratio 6%. After 10 hours of hydrolysis, the enzyme was inactivated in water bath (90°C, 15 min), cooling to room temperature, the solution was centrifuged at 8694×g (4°C, 45 min), and the supernatant was collected as SPPH.

The protein content was measured by Bio-Rad protein assay. The distribution of molecular weight was analyzed by HPLC method, the composition of amino acids was determined by Amino Acids Autoanalyzer (A300 MembraPure, Germany).

### Animal experiments and assays

C57BL/6J mice (4–5 weeks old, weighing 18–20 g, n = 60) were purchased from Huafukang Biotechnology Co. Ltd, Beijing, China. All experimental procedures, approved by Jinan University Committee for Animal Care and Use (No.378653), were performed according to the standard guidelines of animal care and use. Mice were housed at a temperature of 20–26°C and humidity of 50–70% specific pathogen-free environment, with a 12 h light/dark cycle and free access to standard laboratory diet and water. After one week of adaptation period, mice were divided into two groups: normal control group (NC) (n = 10) and model group (n = 50). The normal control group was provided with a normal chow diet, and the model group was maintained on high-fat diet (HFD) (Table 1). The normal chow diet was comprised of food with 10% of the calories from fat, while the HFD consisted of food with 45% of the calories from fat. Body weights of animals were measured daily. After three weeks, when the smallest body weight in the HFD group was 20% more of the smallest body weight in the NC group, the average body weights were calculated, and the model was considered to be successfully established. Then, the model group was randomly divided into five groups (n = 6 per group): HFD+DW group, fed with an HFD plus distilled water (10 ml/kg d); HFD+WSP group, fed with an HFD plus whole *Spirulina platensis* (2 g/kg d); HFD+SPP group, fed with an HFD plus *Spirulina platensis* protein (2 g/kg d); HFD+SPPH group, fed with *Spirulina platensis* protein hydrolysate (2 g/kg d); HFD+SIM group, fed with Simvastatin (10 mg/kg d) as a positive drug group due to the fact that Simvastatin is a lipid-lowering compound and has been shown to exert anti-obesity effects on HFD-fed mice [7]. Each group of mice were kept in two cages (3 mice/cage). The dose 2 g/kg d was based on previous report [21]. The same dose was used for WSP, SPP and SPPH, not based on their protein contents. The reason was that WSP may contain other anti-obesity ingredients except for proteins, the purpose of this study was to see which one among WSP, SPP and SPPH was better under the same dose. All of the drugs were suspended or dissolved in distilled water and administered by gavage (0.5 ml) once a day. The same amount of diet (150 g/cage) was supplied, the remaining diet in each cage was weighed every day, and fresh diets were supplemented. The food intake was calculated as bellows: Mean daily food intake (g/day/mice) = (quantity of diet supplied (150 g)—quantity of remaining diet after 24 h)/amount of mice in each cage. The body weight was recorded weekly. The experimental period lasted for 4 weeks. After an overnight fast, the animals were anesthetized using intraperitoneal (i.p.) sodium pentobarbital (0.06 mg/kg) and kept under anesthesia by infusing (i.p.) sodium pentobarbital (0.020 mg/kg/h) for 3 h, then, under deep anesthesia, all animals were euthanized by cervical dislocation. Blood samples were collected from their hearts by cardiac puncture. Plasma was separated by centrifugation at 1600g for 10 min and tested for serum glucose, TC, TG, HDL-C, LDL-C using commercial assay kits. Liver, brain, spleen and kidney were collected, weighed, snap frozen in liquid nitrogen and stored at −80°C for further analysis. The inhibitory rate of body weights were determined as the formula: body weight inhibition (%) = ((W1-W0)-(W-W0))/(W1-W0)×100%, where W1 is body weight of model control HFD+DW group, W0 is body weight of normal control (NC) group, W is body weight of drug group (HFD+WSP, HFD+SPP, HFD+SPPH or HFD+SIM). Viscera index = viscera weight/mice weight.

**Table 1:** Composition of high fat and control diets.

Ingredient	High fat diet (g/kg)
Casein	200
L-cystein	3
Corn starch	72.8
Maltodextrin	100
Sucrose	172.8
Cellulose	50
Soybean oil	25
Lard	177.5
Minerals AIN-93	35
Vitamins AIN-93	10
Choline bitartrate	2.5
Ingredient	Control diet (g/kg)
Water	79
Fat	45
Ash	67
Cellulose	38
Protein	199
Phosphor	8.9
Calcium	12
Lysine	8.9
Methioninum	1.7
Cysteine	1.2

### RT-PCR array analysis and network mapping

Gene expression profiling was performed using a similar method with slight modification as described before [22]. Specifically, four tissue samples were analyzed: Brain+HFD+DW and Liver+HFD+DW represents the brain and liver tissues of mice from the control group (HFD+DW), respectively; Brain+HFD+SPPH and Liver+HFD+SPPH corresponds to the brain and liver tissues of mice from the hydrolysate group (HFD+SPPH), respectively. Total RNA was isolated from these tissue samples using TRIZOL (Invitrogen, Carlsbad, Calif., USA) method according to the manufacturer’s instructions. The extracted RNA was reversed transcribed using an RT-PCR kit (catalog#CTB101, Chutian Biosciences, China) on the ABI 9700 thermocycler (ABI, Foster City, CA). Self-selected 44 genes related to obesity and house-keeping genes (**S1 Table**) were analyzed by PCR array in 384-well plates with a LightCycler 480 PCR system (Roche Diagnostics, Mannheim, Germany). Amplification was performed as below: pre-denaturation/enzyme inactivation at 94°C for 10 min and denaturation at 94°C for 15 s, followed by 45 cycles of annealing at 60°C for 15 s and extension at 72°C for 20 s. Gene expression was normalized to the mean of all house-keeping genes in the array. Relative changes in gene expressions were calculated using a ΔΔCt (threshold cycle) method, and fold-change values were calculated using the formula of 2^-ΔΔCt^. The genes with more than two-fold changes in gene expression or significant expressions (p < 0.05) by T test were considered as differentially expressed genes. STRING software [23] was employed to implement a network-based analysis of differentially expressed genes.

### Statistical analysis

All experimental results are expressed as the mean ± standard deviation (SD). The student’s-test was performed using SPSS. P-values < 0.05 were considered significant.

## Results

**Fig 1:**
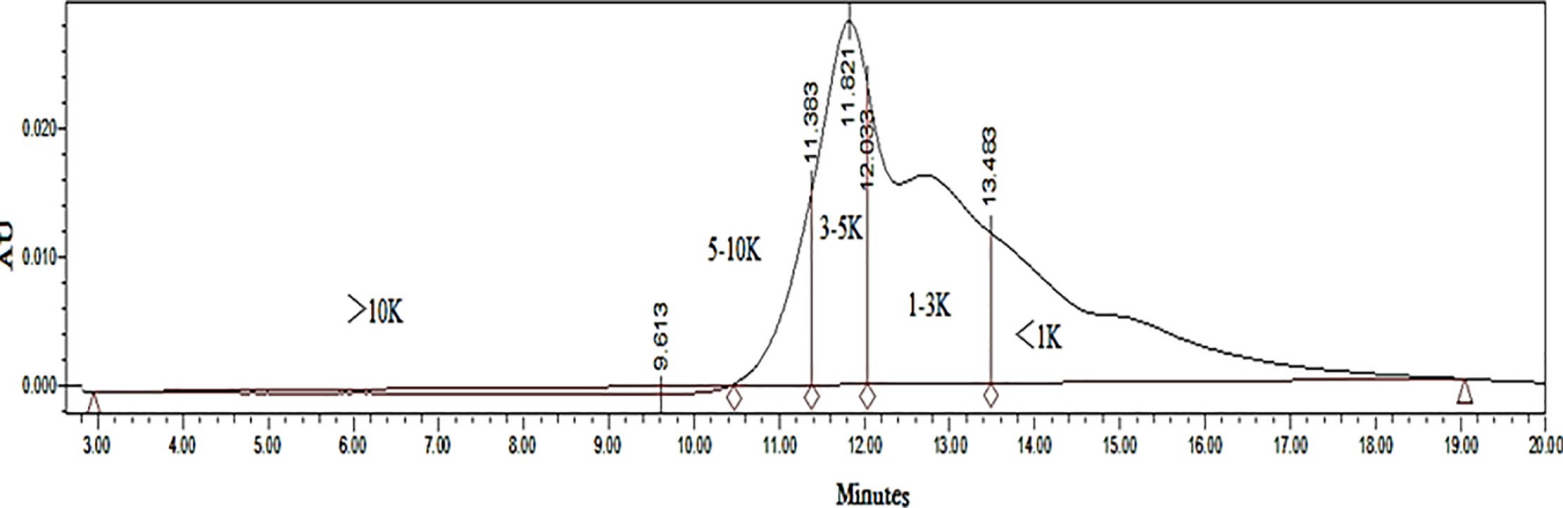
HPLC diagram for molecular weight distribution.

By the combination of freeze-thawing and ultrasonication, 40.7% (w/w) of *Spirulina platensis* proteins were extracted from whole *Spirulina platensis*. Then, pepsin hydrolysis was conducted on the extracted protein to obtain *Spirulina platensis* protein hydrolysate with a 3.6% of hydrolysis degree. Molecular weight distribution by HPLC (Fig 1) showed that the percentages of three fractions (>10K, 3-10K and <3K) in the hydrolysate were determined as 4.4%, 31.7% and 63.9%, respectively, i.e. most of them were small molecule fractions. Their amino acid composition was displayed in S2 Table.

**Fig 2:**
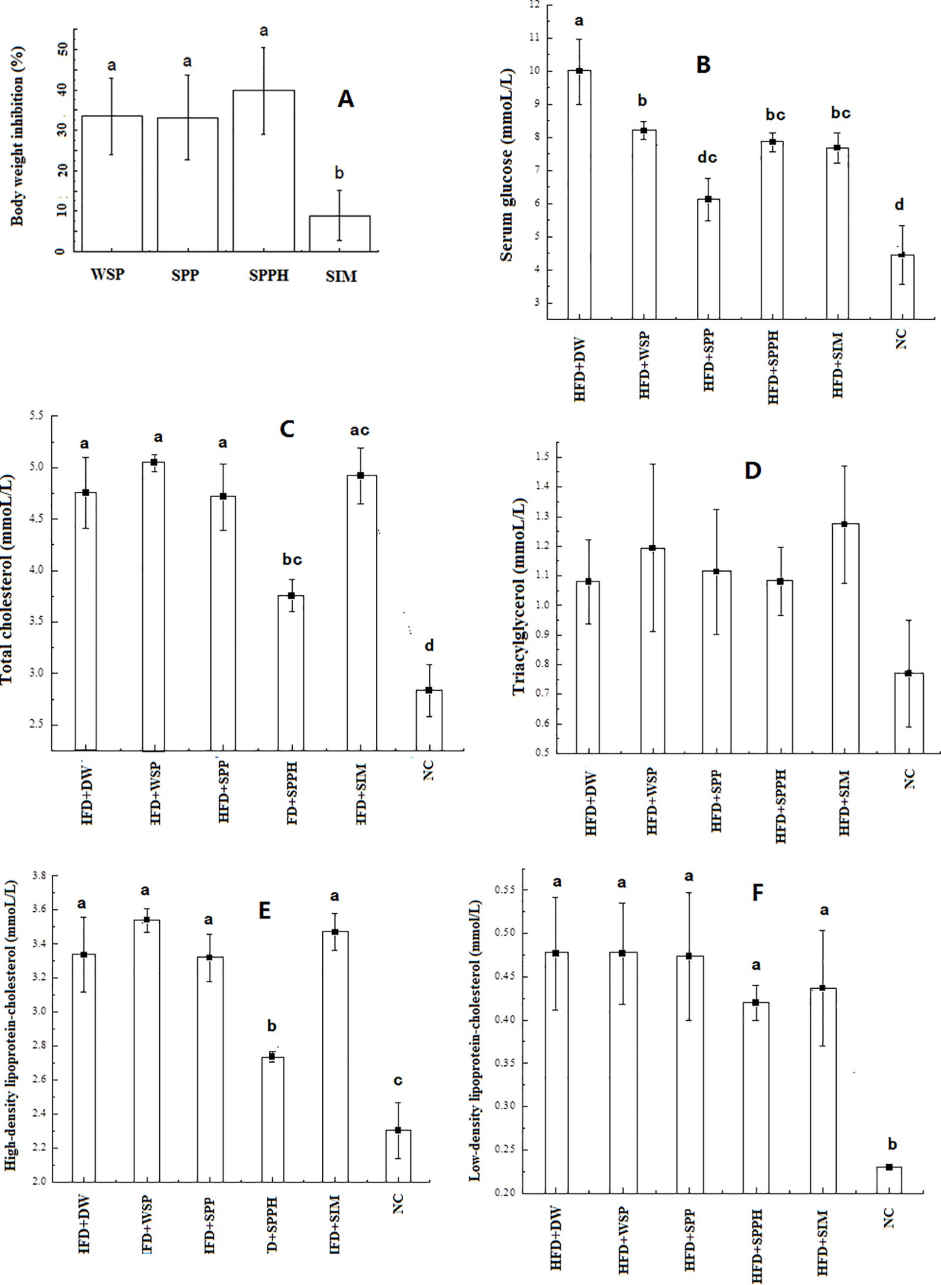
Biochemical assay for anti-obesity effects of whole *Spirulina platensis* (WSP), *Spirulina platensis* protein (SPP), *Spirulina platensis* protein hydrolysate (SPPH) and Simvastatin (SIM) in high-fat diet (HFD) fed mice, (A) body weights, (B) serum glucose, (C) total cholesterol (TC), (D) triacylglycerol (TG), (E) high-density lipoprotein cholesterol and (HDLC), (F) low-density lipoprotein cholesterol (LDLC). DW is distilled water, NC is normal control. Different characters (a,b,c,d) indicated significant difference.

Subsequently, anti-obesity effects of different components were investigated in HFD-fed mice. The body weight changes were shown in Table 2. The results showed that weight reduction percentages of WSP, SPP and SPPH were 33.5%±7.3%、33.1%±9.1% and 39.8%±9.7%, respectively, compared with HFD group (Fig 2A), which were far greater than the percentage of positive drug Simvastatin 8.3%±4.6%. Within four weeks of experimental period, the average food intake for model control group HFD+DW was 3.67±0.25 g/day/mouse, and the average food intake for experimental groups (HFD+WSP, HFD+SPP, HFD+SPPH and HFD+SIM) was 3.78±0.46, 3.67±0.23, 3.5±0.18 and 3.75±0.33 g/day/mouse, respectively, which were statistically not significant (p>0.05) between different groups, meaning that the weight reduction was not caused by inhibiting appetite. The liver, spleen and kidney weights under different treatments were displayed in S1 Fig. The results indicated that all treatments had no significant differences (p>0.05), i.e. Spirulina platensis treatments had no significant influences (p>0.05) on liver, spleen and kidney, compared with normal control group.

**Table 2:** The body weight changes of mice.

Group	n	Initial weight (g)	Weight after model establishment (g)	Final weight (g)
HFD+DW	6	23.97±0.9865 a	28.73±0.602771 a	30.37±1.40119
HFD+WSP	6	23.72±1.225561 a	28.62±0.349285 a	28.48±1.384558
HFD+SPP	6	23.32±0.870057 a	29±0.804156 a	28.5±1.208305
HFD+SPPH	6	23.5±1.115796 a	28.6±0.2 a	28.13±0.648074
HFD+SIM	6	23.43±0.805709 a	28.67±0.51316 a	29.87±1.106044
NC	6	23.1±0.547723 a	24±0.754983 b	24.73±0.52915

Note: different characters (a,b) indicated significant difference (p<0.05)

Serum glucose assay (Fig 2B) indicated that high-fat diet greatly elevated serum glucose content up to 10.1 ± 1.01 mmol/L (HFD+DW group), while *Spirulina platensis* intervention remarkedly reduced serum glucose content down to 8.2±0.45, 6.1±0.64, 7.7±0.36 and 7.6±0.89 mmol/L for HFD+WSP, HFD+SPP, HFD+SPPH and HFD+SIM, respectively, which were higher than normal control (NC) group (4.5±0.56 mmol/L). This implies that whole *Spirulina platensis*, *Spirulina platensis* protein, *Spirulina platensis* protein hydrolysate and Simvastatin can lower serum glucose level by 18.8%±1.3%, 39.6%±2.4%, 23.8%±1.6% and 24.8%±1.9%, respectively, compared with HFD groups, but can’t restore to normal level. Among them, *Spirulina platensis* protein was best, *Spirulina platensis* protein hydrolysate was close to Simvastatin.

High-fat diet elevated total cholesterol (TC) content (4.8±0.79 mmol/L), compared with NC group (2.7±0.43 mmol/L) (Fig 2C). However, WSP, SPP and Simvastatin did not inhibit such an elevation, in which TC contents were up to 5.1±0.17, 4.7±0.62 and 4.9±0.59 mmol/L, respectively. SPPH significantly (p<0.05) reduced such an elevation, TC content was down to 3.8±0.42 mmol/L, although still higher than NC group. Thus, whole *Spirulina platensis*, *Spirulina platensis* protein and positive drug Simvastatin can’t reduce TC content, only *Spirulina platensis* protein hydrolysate can lower TC level by 20.8%±1.4%, compared with HFD group.

Fig 2D showed that triglyceride contents were not statistically significant changed (p>0.05) in all HFD groups (HFD+DW (1.08±0.12 mmol/L), HFD+WSP (1.18±0.26 mmol/L), HFD+SPP (1.11±0.17 mmol/L), HFD+SPPH (1.07±0.11 mmol/L) and HFD+SIM (1.26±0.15 mmol/L)), compared with NC group (0.74±0.14 mmol/L), i.e. all *Spirulina platensis* interventions including positive drug Simvastatin had no significant (p>0.05) influence on triglyceride content in present experiment.

Similarly, high-fat diet significantly (p<0.05) elevated the contents of high-density lipoprotein cholesterol (HDLC) and low-density lipoprotein cholesterol (LDLC), compared with normal control (Fig 2E and 2F). SPPH significantly (p<0.05) reduced the increases of HDLC caused by HFD, WSP and Simvastatin altered HDLC contents but not significantly (p>0.05); SPPH and Simvastatin influenced LDLC but not significantly (p>0.05).

**Fig 3:**
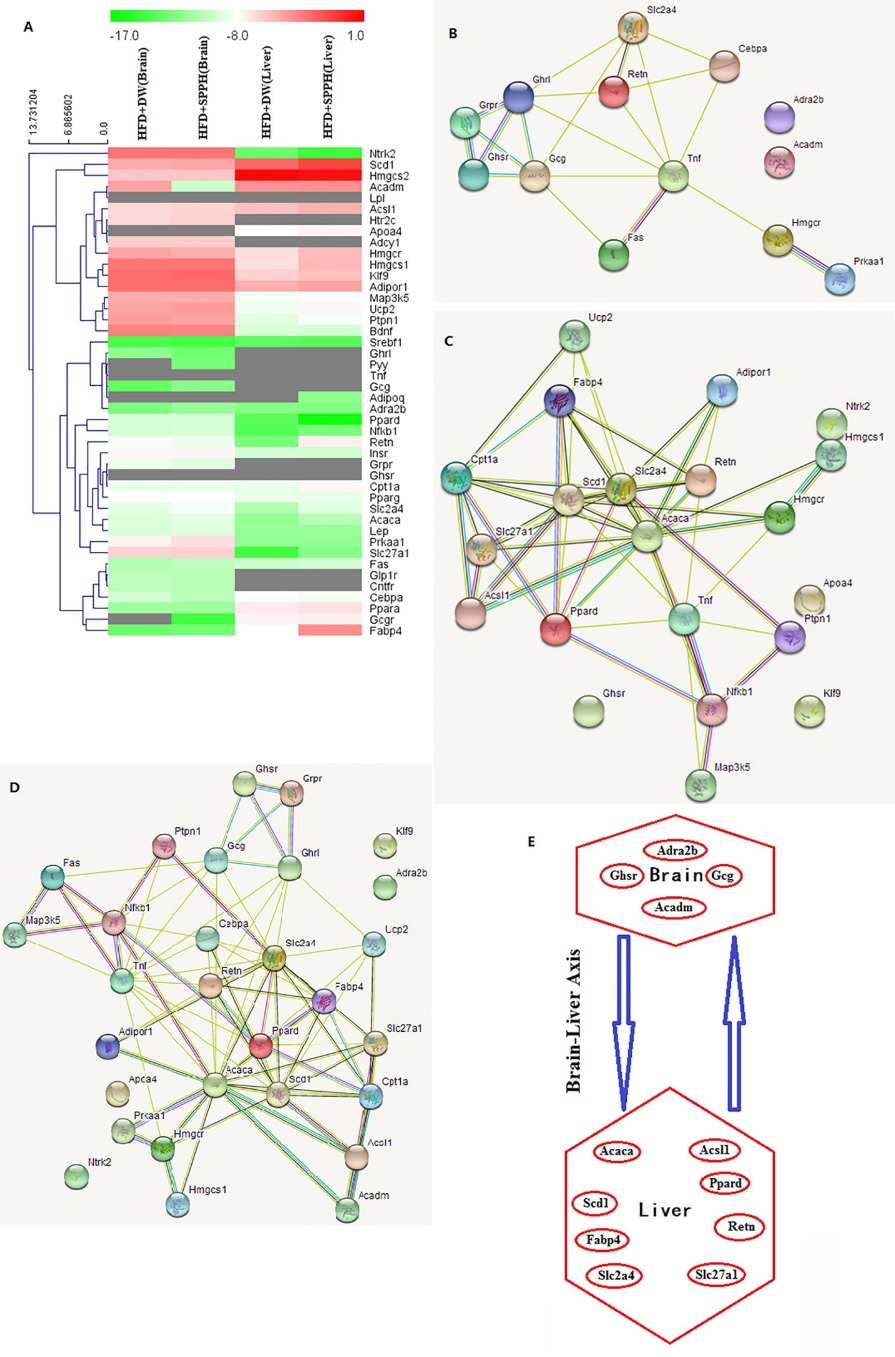
(A) Heatmap of gene expression for four groups: high-fat diet (HFD)+distilled water (DW) (brain), high-fat diet (HFD)+ *Spirulina platensis* protein hydrolysate (SPPH) (brain), high-fat diet (HFD)+distilled water (DW) (liver), high-fat diet (HFD)+ *Spirulina platensis* protein hydrolysate (SPPH) (liver). (B) The interaction network mapped by significantly expressed genes in brain tissue. (C) The interaction network mapped by significantly expressed genes in liver tissues. (D) The interaction network mapped by significantly expressed genes in brain and liver tissues. (E) Proposed brain-liver axis acted by *Spirulina platensis* protein hydrolysate (SPPH).

In order to understand anti-obesity mechanism of SPPH, gene expression analysis was conducted on brain and liver tissues of mice by using self-designed RT-PCR array. The gene changes in brain tissues of high fat diet fed-mice treated with distilled water and *Spirulina platensis* protein hydrolysate were displayed in S3 Table. In total, 22 genes were up-regulated and 17 genes were down-regulated. Among them, the most significantly (p<0.005) changed gene was Acadm (-34.7 fold), other top changed genes include: Gcg (2.5 fold), Adra2b (2 fold), Ghsr (2 fold), Cebpa (-1.8 fold), Prkaa1 (1.6 fold), Slc2a4 (1.5 fold). Likewise, the gene changes in liver tissues of high fat diet fed-mice treated with distilled water and *Spirulina platensis* protein hydrolysate were summarized in S4 Table. There were 23 up-regulated genes and 10 down-regulated genes. The most markedly changed genes were Retn (39 fold) and Fabp4 (15.5 fold), other top changed genes include: Ppard (6 fold), Slc27a1 (5.4 fold), Slc2a4 (3.3 fold), Ntrk2 (-3.1 fold), Hmgcs1 (2.7 fold), Nfkb1 (2.4 fold), Scd1 (2.4 fold), Hmgcr (2.3 fold) and Cpt1a (2 fold). The heatmap of gene expression in brain and liver tissues was shown in Fig 3A. Obviously, different gene expression pattern was observed in brain and liver tissues. Generally, the gene changes in liver tissue were greater than the gene changes in brain tissue, after SPPH intervention.

By STRING analysis, the interaction networks of significantly expressed genes were identified. Fig 3B displayed the interaction network mapped by significantly expressed genes in brain tissue. The top pathways include: Non-alcoholic fatty liver disease (NAFLD) (Fas, Tnf, Cebpa,Prkaa1), Adipocytokine signaling pathway (Slc2a4, Tnf, Prkaa1), AMPK signaling pathway (Slc2a4, Hmgcr, Prkaa1), Type II diabetes mellitus (Slc2a4, Tnf). The interaction network mapped by significantly expressed genes in liver tissue was shown in Fig 3C. The top pathways include: PPAR signaling pathway (Acsl1, Slc27a1, Cpt1a, Ppard, Scd1, Fabp4), Adipocytokine signaling pathway (Acsl1, Cpt1a, Scd1, Adipor1, Tnf, Nfkb1), AMPK signaling pathway (Cpt1a, Scd1, Slc2a4, Acaca, Hmgcr), Fatty acid metabolism (Acsl1, Cpt1a, Scd1, Acaca), Non-alcoholic fatty liver disease (NAFLD)(Adipor1, Tnf, Nfkb1, Map3k5). Furthermore, combining the significantly expressed genes in brain and liver tissues, a systematic network was mapped (Fig 3D). The top pathways were as below: PPAR signaling pathway (Ppard, Fabp4, Scd1, Slc27a1, Cpt1a, Acsl1, Acadm), Adipocytokine signaling pathway (Prkaa1, Adipor1, Tnf, Nfkb1, Slc2a4, Cpt1a, Acsl1), AMPK signaling pathway (Prkaa1, Hmgcr, Adipor1, Acaca, Scd1, Cpt1a, Slc2a4), Non-alcoholic fatty liver disease (NAFLD) (Prkaa1, Adipor1, Tnf, nfkb1, Cebpa, Map3k5, Fas), Fatty acid metabolism (Acaca, Scd1, Cpt1a, Acsl1, Acadm), MAPK signaling pathway (Tnf, Nfkb1, Map3k5, Fas, Ntrk2), TNF signaling pathway (Tnf, Nfkb1, Map3k5, Fas), Insulin signaling pathway (Prkaa1, Acaca, Slc2a4, Ptbn1).

## Discussion

By comparing anti-obesity effects of WSP, SPP and SPPH with DW in high fat diet-fed mice, the results demonstrated that SPPH was best for weight reduction (39.8%) and total cholesterol reduction (20.8%), while SPP was best for lowering glucose (39.6%), among WSP, SPP and SPPH. So, SPP and SPPH were superior to WSP, SPPH was slightly better that SPP. Thus, the same dose but different effects were observed for WSP, SPP and SPPH, this may be explained by the compositional difference in them: WSP contains nonprotein components, SPP is large molecule protein, and SPPH primarily consists of small molecular peptides. In addition, WSP, SPP and SPPH had no significant influences on triglyceride and LDLC. However, SPPH unexpectedly decreased HDLC, which was called “good” cholesterol and is considered to lower the risk for heart disease and stroke. Currently, there is no strong clinical evidence to show that high plasma HDLC is associated with reduced cardiovascular events [24–26]. Studies reported that orlistat decreased HDL-3 subclasses in obese hyperlipidemic patients [27], and also decreased small HDL subclasses in obese metabolic syndrome patients [28]. Similarly, ezetimibe was reported to reduce small HDL subfractions in patients with primary dyslipidaemia [29]. Thus, the anti-obesity effect of SPPH could have some similar mechanism of action with these anti-obesity drugs in decreasing HDLC. Notably, the reduction in HDLC may not be harmful, meta-analysis indicated that increasing HDLC levels were not associated with reduced all-cause mortality, coronary heart disease mortality, myocardial infarction, or stroke [30]. In fact, very high HDL-C level was associated with increased risk of all-cause death [31].

Studies indicated that a gut-brain–liver axis has been considered to play an important role in obesity and diabetes [32–34]. In this study, the gene changes in brain and liver tissues of SPPH-treated obese mice were simultaneously investigated. It is very clear that brain and liver tissues displayed different gene expression patterns after SPPH intervention. Such as, the most significantly changed gene in brain was Acadm (-34.7 fold), while it was slightly increased in liver (1.08 fold); the genes with more than 5-fold changes in liver include Retn (39 fold), Fabp4 (15.5 fold), Ppard (6 fold) and Slc27a1 (5.4 fold), but they were not significantly changed in brain: Retn (-1.39 fold), Fabp4 (1.23 fold), Ppard (-1.02 fold) and Slc27a1 (1.1 fold), respectively. Previous studies indicated that green tea extract suppressed adiposity in diet-induced obese zebrafish by increasing the expression of lipid catabolism genes Acox1 (acyl-coenzyme A oxidase 1, palmitoyl) and Acadm (acyl-coenzyme A dehydrogenase) [35]. This is contrary to our result in brain but consistent with our result in liver. Ohashi et al. [36] reported that the anti-obesity effect of conjugated linoleic acids was associated with decreased resistin. Hwang et al. [37] investigated the anti-obesity and anti-diabetic effects of deep sea water (DSW) in ob/ob mice, they found that plasma protein level of resistin was decreased in adipose tissue of DSW-fed mice. A recent report also indicated that isothiocyanate-rich Moringa oleifera extract reduced weight gain, insulin resistance, and hepatic gluconeogenesis in mice by reducing plasma insulin, leptin and resistin [38]. These changes of Retn in adipose tissue or serum were inconsistent with our result in liver but consistent with our result in brain. Moon et al. [39] showed that anti-obesity effects of quercetin-rich onion peel extract were followed by up-regulation of fatty acid binding protein 4 (Fabp4) in high fat-fed rats. Yang et al. [40] demonstrated that weight loss herbal intervention therapy (W-LHIT) reduced body weight, without suppression of appetite, in obese mice, by significantly increasing the gene expression of Pparg (peroxisome proliferator activated receptor gamma) and Fabp4 (fatty acid binding protein 4) epdidymal fat tissues. In contrast, Lee et al. [41] Monascus pilosus-fermented black soybean inhibits lipid accumulation in adipocytes and in high-fat diet-induced obese mice, but significantly lower gene expression of adipogenesis-related genes like peroxisome proliferator-activated receptor gamma (Pparg) and fatty acid-binding protein 4 (Fabp4). Kim et al. [42] showed that anti-obesity effect of Solidago virgaaurea extract in high-fat diet-fed SD rat was associated with decreased acetyl-CoA carboxylase, fatty acid synthase, and Fabp4. Tsai et al. [43] reported that anti-adipogenesis effect of raspberry ketone was mediated by reducing peroxisome proliferation-activated receptor-gamma (Pparg), fatty acid synthase (Fas), and fatty acid-binding protein 4 (Fabp4) expressions in 3T3-L1 cells. These inconsistent results reflect their different anti-obesity mechanisms. Hence, the changes in gene expression caused by anti-obesity agents are drug or supplement-dependent and tissue-dependent, highlights the importance of cooperation among organs or gut-brain-liver axis to some extents.

Network analysis demonstrated that the significantly expressed genes in brain tissue were mapped into a network with core genes Tnf, Gcg and Ghrl, each of them had 6 connection edges (Fig 3B). The main pathways include Non-alcoholic fatty liver disease (NAFLD), Adipocytokine signaling pathway and AMPK signaling pathway. Similarly, the significantly expressed genes in liver tissue were mapped into a network with core genes Acaca, Slc2a4 and Scd1 (Fig 3C). The main pathways include PPAR signaling pathway, Adipocytokine signaling pathway, AMPK signaling pathway, Fatty acid metabolism, Non-alcoholic fatty liver disease (NAFLD). Surprisingly, the most significantly changed gene Adcadm (-34.7 fold) in brain existed beyond the gene network of brain as an individual component. Maybe this suggests that the major interacting target of Acadm was not in brain but in other organs such as liver in response to SPPH intervention. Indeed, by taking the significantly changed genes in brain and liver tissues into consideration as a whole, Acadm was mapped into a network with direct link to Acaca and Acsl1 (Fig 3D), suggesting that probably there is direct interaction between Acadm in brain and Acaca as well as Acsl1 in liver after SPPH intervention, i.e. acting on brain-liver axis. A speculative mechanism of action for SPPH is proposed in Fig 3E. However, the detailed mechanisms, specially the communication between brain and liver, the messenger molecules between Acadm and Acaca or Acsl1, deserve intensive research in the future.

In a word, the strong points of this study are that the anti-obesity effect of *Spirulina platensis* protein hydrolysate (SPPH) was explored in terms of brain-liver axis for the first time, different gene expression patterns were observed in response to intervention of SPPH, and different core regulatory networks were identified for the action of SPPH on brain and liver.

## Conclusion

The anti-obesity effects of Spirulina protein (SPP) or peptide (SPPH) are superior to than the whole Spirulina (WSP), SPPH is slightly better than SPP, under the same dose. SPPH had good glucose reduction, weight reduction and total cholesterol reduction activities by modulating the expressions of some key genes in brain and liver, such as Acadm, Retn, Fabp4, Ppard, Slc27a1, etc. These genes have been shown to be associated with lipid metabolism and accumulation in literature, but the detailed link of their changes to glucose or weight reduction requires further study.

## Supporting information

S1 Checklist Animal research: Reporting in vivo experiments. (DOCX)

S1 Table Self-selected and house-keeping genes for PCR array. (DOCX)

S2 Table Amino acids composition of Spirulina platensis protein hydrolysate. (DOCX)

S3 Table Gene changes in brain tissues of high fat diet fed-mice treated with distilled water and Spirulina platensis protein hydrolysate. (DOCX)

S4 Table Gene changes in liver tissues of high fat diet fed-mice treated with distilled water and Spirulina platensis protein hydrolysate. (DOCX)

S1 Fig The liver, spleen and kidney coefficients under different treatments. (DOCX)
