# Supplementary material for: Anti-obesity effects of Spirulina platensis protein hydrolysate by modulating brain-liver axis in high-fat diet fed mice
Source: PLoS One. 2019 Jun 20;14(6):e0218543. doi: 10.1371/journal.pone.0218543 (PMC6586325; doi:10.1371/journal.pone.0218543)

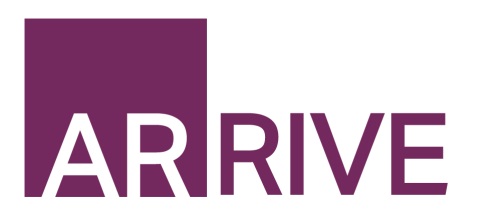


The ARRIVE Guidelines Checklist

Animal Research: Reporting In Vivo Experiments

Carol Kilkenny^1^, William J Browne^2^, Innes C Cuthill^3^, Michael Emerson^4^ and Douglas G Altman^5^

*^1^The National Centre for the Replacement, Refinement and Reduction of Animals in Research, London, UK, ^2^School of Veterinary Science, University of Bristol, Bristol, UK, ^3^School of Biological Sciences, University of Bristol, Bristol, UK, ^4^National Heart and Lung Institute, Imperial College London, UK, ^5^Centre for Statistics in Medicine, University of Oxford, Oxford, UK.*

|  | | ITEM | RECOMMENDATION | Section/ Paragraph |
| --- | --- | --- | --- | --- |
| 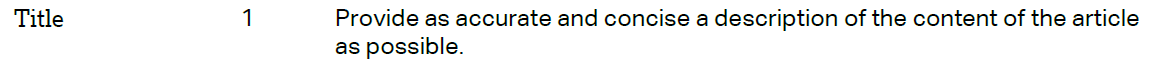 | | | Title |  |
| 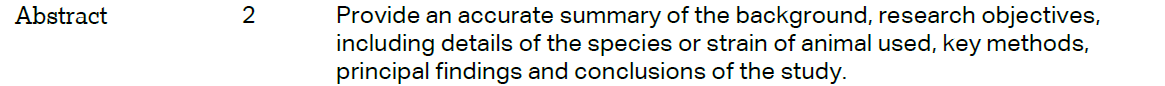 | | | Abstract |  |
| INTRODUCTION | | |  |  |
| 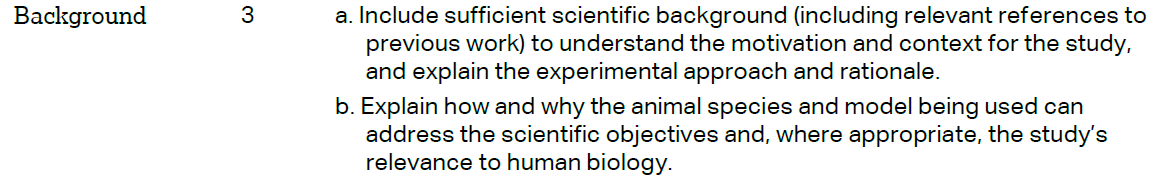 | | | Paragraph 1-4 |  |
| 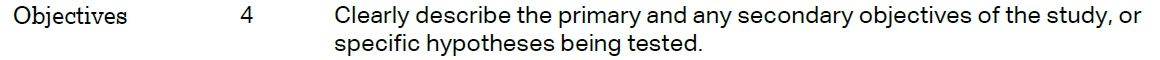 | | | Paragraph 4 |  |
| METHODS | | |  |  |
| 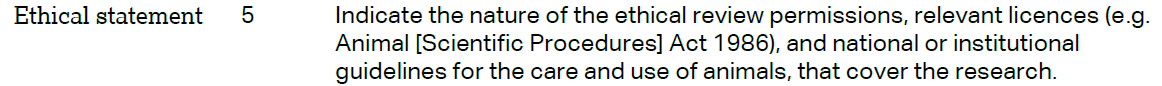 | | | Paragraph 6 |  |
| 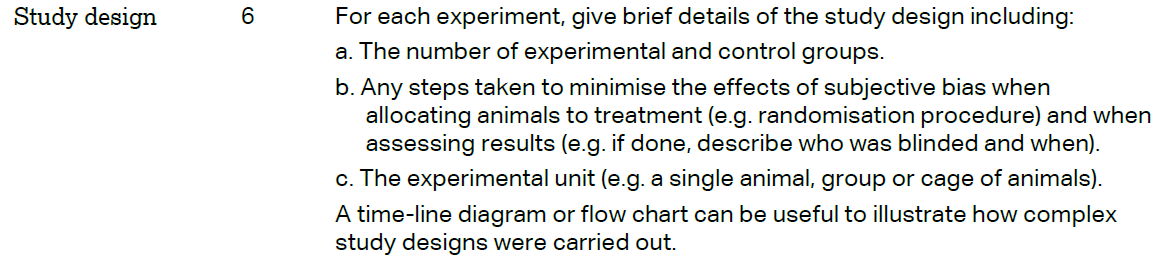 | | | Paragraph 6 |  |
| 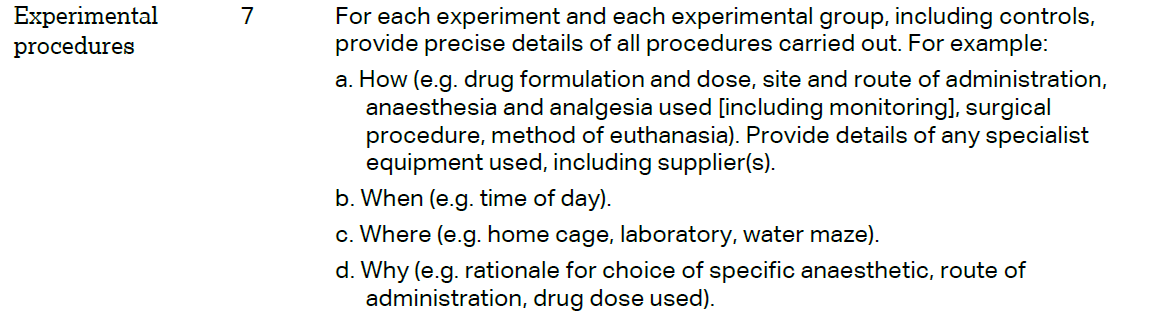 | | | Paragraph 6 |  |
| 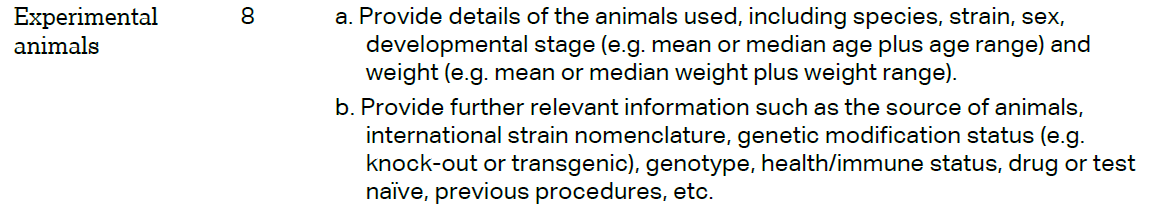 | | | Paragraph 6 |  |

The ARRIVE guidelines. Originally published in *PLoS Biology*, June 2010^1^

| 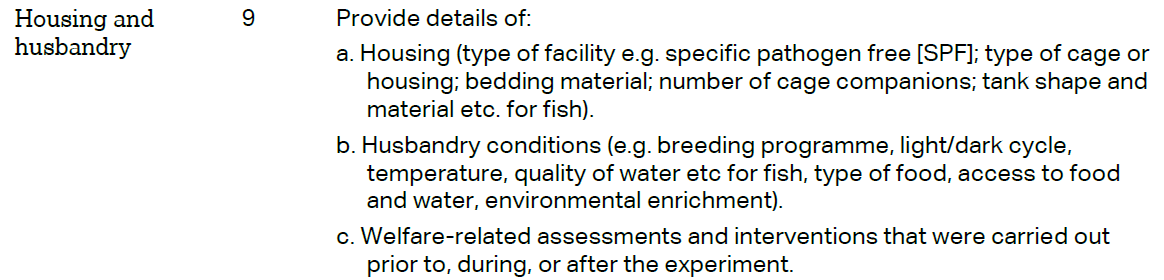 | Paragraph 6 | |
| --- | --- | --- |
| 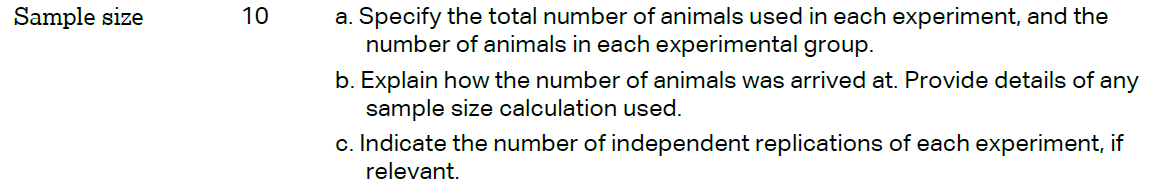 | Paragraph 6 | |
| 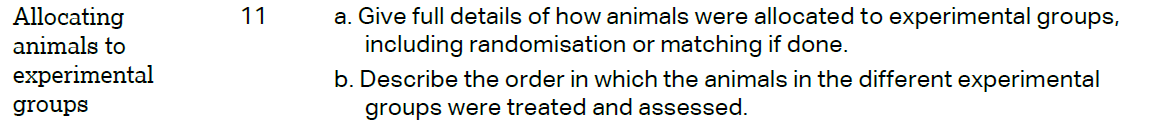 | Paragraph 6 | |
| 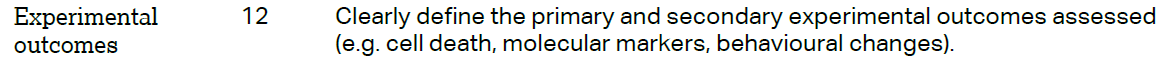 | Paragraph 6 | |
| 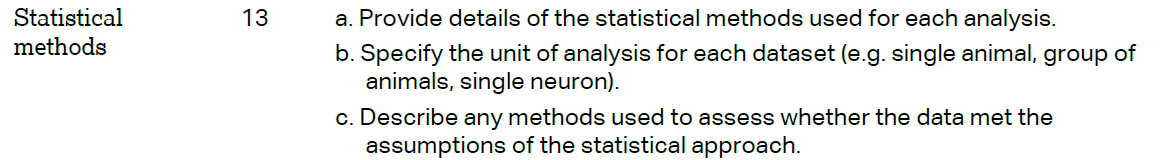 | Paragraph 8 | |
| RESULTS |  | |
| 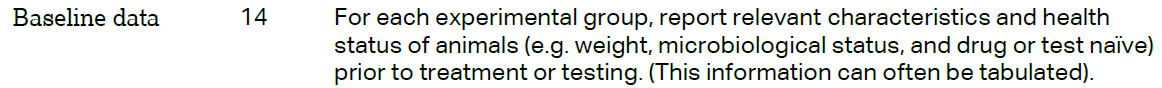 | Methods Paragraph 6 | |
| 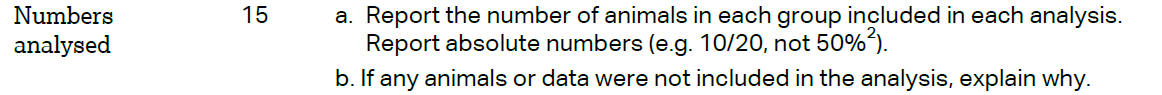 | Methods Paragraph 6 | |
| 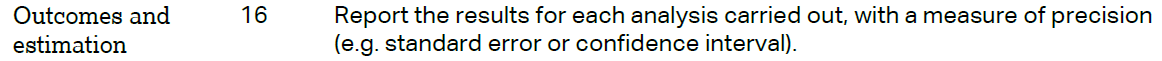 | Paragraph 2-6 | |
| 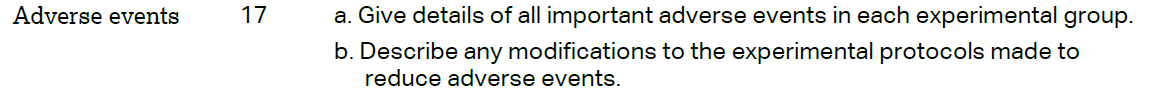 | No adverse events | |
| DISCUSSION |  | |
| 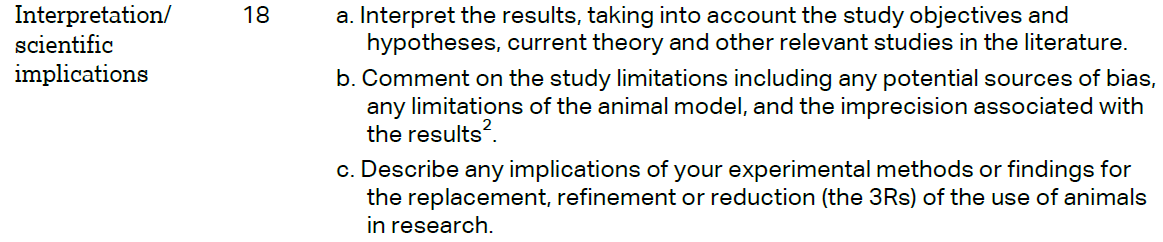 | Paragraph 1-4 | |
| 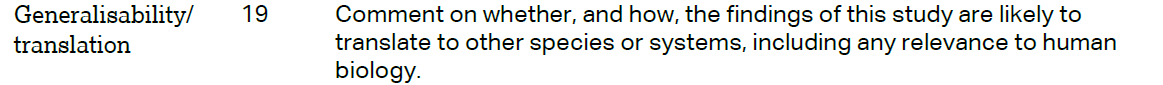 | Paragraph 1-4 | |
| 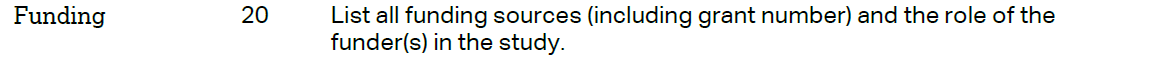 | | No specific funding |


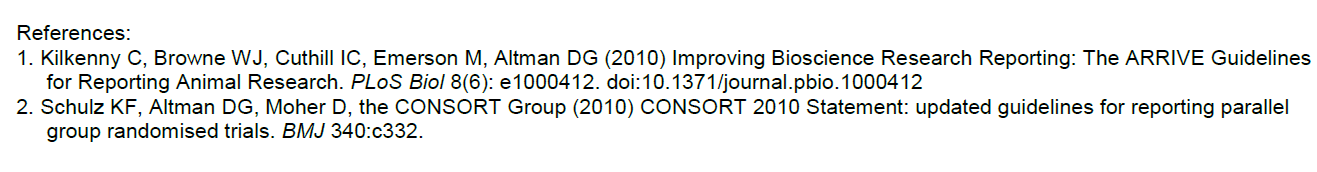

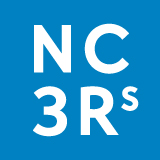

Supplement: S1 Checklist — (DOCX) [file pone.0218543.s001.docx]
