# Supplementary material for: Anti-obesity effects of Spirulina platensis protein hydrolysate by modulating brain-liver axis in high-fat diet fed mice
Source: PLoS One. 2019 Jun 20;14(6):e0218543. doi: 10.1371/journal.pone.0218543 (PMC6586325; doi:10.1371/journal.pone.0218543)
Supplement: S1 Table — (DOCX) [file pone.0218543.s002.docx]

S1 Table Self-selected and house-keeping genes for PCR array

| **No** | **Gene symbol** | **Gene name** | **Pathways involved** |
| --- | --- | --- | --- |
| 1 | Acadm | acyl-Coenzyme A dehydrogenase, medium chain | PPAR signaling pathway, Fatty acid metabolism |
| 2 | Adipoq | adiponectin, C1Q and collagen domain containing | PPAR signaling pathway, AMPK signaling pathway, Non-alcoholic fatty liver disease, Adipocytokine signaling pathway, Type II diabetes mellitus |
| 3 | Cpt1a | carnitine palmitoyltransferase 1a, liver | PPAR signaling pathway, AMPK signaling pathway, Adipocytokine signaling pathway, Fatty acid metabolism |
| 4 | Gcg | glucagon | G-protein coupled receptor signaling pathway |
| 5 | Htr2c | 5-hydroxytryptamine (serotonin) receptor 2C | Neuroactive ligand-receptor interaction, Calcium signaling pathway |
| 6 | Klf9 | Kruppel-like factor 9 | Zinc finger transcription factor |
| 7 | Srebf1 | sterol regulatory element binding transcription factor 1 | AMPK signaling pathway, Non-alcoholic fatty liver disease, Insulin signaling pathway |
| 8 | Ucp2 | uncoupling protein 2 (mitochondrial, proton carrier) | Insulin signaling pathway |
| 9 | Adipor1 | adiponectin receptor 1 | AMPK signaling pathway, Non-alcoholic fatty liver disease, Adipocytokine signaling pathway |
| 10 | Prkaa1 | protein kinase, AMP-activated, alpha 1 catalytic subunit | AMPK signaling pathway, Non-alcoholic fatty liver disease, Adipocytokine signaling pathway, Insulin signaling pathway, FoxO signaling pathway, mTOR signaling pathway |
| 11 | Ppard | peroxisome proliferator activator receptor delta | PPAR signaling pathway |
| 12 | Pparg | peroxisome proliferator activated receptor gamma | PPAR signaling pathway, AMPK signaling pathway |
| 13 | Ntrk2 | neurotrophic tyrosine kinase, receptor, type 2 | MAPK signaling pathway, Neurotrophin signaling pathway |
| 14 | Bdnf | brain derived neurotrophic factor | MAPK signaling pathway, Neurotrophin signaling pathway |
| 15 | Lpl | lipoprotein lipase | PPAR signaling pathway |
| 16 | Cebpa | CCAAT/enhancer binding protein (C/EBP), alpha | Non-alcoholic fatty liver disease |
| 17 | Adra2b | adrenergic receptor, alpha 2b | Neuroactive ligand-receptor interaction, cGMP-PKG signaling pathway |
| 18 | Fabp4 | fatty acid binding protein 4, adipocyte | PPAR signaling pathway |
| 19 | Gcgr | glucagon receptor | Neuroactive ligand-receptor interaction, G-protein coupled receptor signaling pathway |
| 20 | Glp1r | glucagon-like peptide 1 receptor | Neuroactive ligand-receptor interaction |
| 21 | Lep | leptin | AMPK signaling pathway, Non-alcoholic fatty liver disease, Adipocytokine signaling pathway, Neuroactive ligand-receptor interaction |
| 22 | Ppara | peroxisome proliferator activated receptor alpha | PPAR signaling pathway, Non-alcoholic fatty liver disease, Adipocytokine signaling pathway |
| 23 | Map3k5 | mitogen-activated protein kinase kinase kinase 5 | Non-alcoholic fatty liver disease, MAPK signaling pathway, Neurotrophin signaling pathway |
| 24 | Slc27a1 | solute carrier family 27 (fatty acid transporter), member 1 | PPAR signaling pathway |
| 25 | Acaca | acetyl-Coenzyme A carboxylase alpha | AMPK signaling pathway, Fatty acid metabolism, Insulin signaling pathway |
| 26 | Ghsr | growth hormone secretagogue receptor | Neuroactive ligand-receptor interaction |
| 27 | Scd1 | stearoyl-Coenzyme A desaturase 1 | PPAR signaling pathway, AMPK signaling pathway, Fatty acid metabolism |
| 28 | Nfkb1 | nuclear factor of kappa light polypeptide gene enhancer in B cells 1, p105 | Non-alcoholic fatty liver disease, Adipocytokine signaling pathway, MAPK signaling pathway, Neurotrophin signaling pathway, NOD-like receptor signaling pathway |
| 29 | Insr | insulin receptor | AMPK signaling pathway, Non-alcoholic fatty liver disease, Insulin signaling pathway, Type II diabetes mellitus, FoxO signaling pathway, cGMP-PKG signaling pathway |
| 30 | Hmgcs1 | 3-hydroxy-3-methylglutaryl-Coenzyme A synthase 1 | Valine, leucine and isoleucine degradation |
| 31 | Apoa4 | apolipoprotein A-IV | Gut Hormones and Receptors, Fat digestion and absorption |
| 32 | Cntfr | ciliary neurotrophic factor receptor | Cytokine-cytokine receptor interaction |
| 33 | Grpr | gastrin releasing peptide receptor | Neuroactive ligand-receptor interaction, Calcium signaling pathway |
| 34 | Hmgcs2 | 3-hydroxy-3-methylglutaryl-Coenzyme A synthase 2 | PPAR signaling pathway |
| 35 | Ptpn1 | protein tyrosine phosphatase, non-receptor type 1 | Insulin signaling pathway |
| 36 | Slc2a4 | solute carrier family 2 (facilitated glucose transporter), member 4 | AMPK signaling pathway, Adipocytokine signaling pathway, Insulin signaling pathway, Type II diabetes mellitus, FoxO signaling pathway |
| 37 | Retn | resistin | Insulin signaling pathway |
| 38 | Ghrl | ghrelin | Gut Hormone and Receptor |
| 39 | Pyy | peptide YY | Gut Hormone and Receptor |
| 40 | Adcy1 | adenylate cyclase 1 | cGMP-PKG signaling pathway, Calcium signaling pathway, Bile secretion |
| 41 | Fas | Fas (TNF receptor superfamily member 6) | Non-alcoholic fatty liver disease, MAPK signaling pathway |
| 42 | Tnf | tumor necrosis factor | Non-alcoholic fatty liver disease, Adipocytokine signaling pathway, Type II diabetes mellitus, MAPK signaling pathway, mTOR signaling pathway, NOD-like receptor signaling pathway |
| 43 | Acsl1 | acyl-CoA synthetase long-chain family member 1 | PPAR signaling pathway, Adipocytokine signaling pathway, Fatty acid metabolism |
| 44 | Hmgcr | 3-hydroxy-3-methylglutaryl-Coenzyme A reductase | AMPK signaling pathway, Bile secretion |
| 45 | B2m | House-keeping gene |  |
| 46 | Actb | House-keeping gene |  |
| 47 | RTC | Reverse transcription control |  |
| 48 | PPC | Positive proliferation control |  |
