# Supplementary material for: Anti-obesity effects of Spirulina platensis protein hydrolysate by modulating brain-liver axis in high-fat diet fed mice
Source: PLoS One. 2019 Jun 20;14(6):e0218543. doi: 10.1371/journal.pone.0218543 (PMC6586325; doi:10.1371/journal.pone.0218543)
Supplement: S2 Table — (DOCX) [file pone.0218543.s003.docx]

S2 Table Amino acids composition of *Spirulina platensis* protein hydrolysate

| Amino acid name | Whole *Spirulina platensis* (mg/g) | *Spirulina platensis* protein（mg/g） | *Spirulina platensis* protein hydrolysate (mg/g) |
| --- | --- | --- | --- |
| Asp | 49.874 | 51.274 | 41.964 |
| Thr | 25.55 | 26.301 | 22.001 |
| Ser | 24.982 | 25.972 | 20.897 |
| Glu | 85.143 | 86.716 | 71.451 |
| Gly | 26.528 | 27.059 | 20.478 |
| Ala | 39.719 | 40.039 | 31.06 |
| Cys | 2.038 | 2.843 | 4.618 |
| Val | 31.95 | 32.379 | 23.073 |
| Met | 11.369 | 8.363 | 9.75 |
| Ile | 29.886 | 30.154 | 22.189 |
| Leu | 46.518 | 47.393 | 34.4 |
| Tyr | 22.38 | 22.319 | 14.045 |
| Phe | 27.919 | 25.548 | 19.735 |
| His | 10.092 | 10.511 | 8.007 |
| Lys | 25.99 | 25.265 | 21.129 |
| Arg | 31.129 | 28.486 | 21.982 |
| Pro | 20.61 | 19.666 | 15.263 |
