# Supplementary material for: Anti-obesity effects of Spirulina platensis protein hydrolysate by modulating brain-liver axis in high-fat diet fed mice
Source: PLoS One. 2019 Jun 20;14(6):e0218543. doi: 10.1371/journal.pone.0218543 (PMC6586325; doi:10.1371/journal.pone.0218543)
Supplement: S3 Table — (DOCX) [file pone.0218543.s004.docx]

S3 Table Gene changes in brain tissues of high fat diet fed-mice treated with distilled water and *Spirulina platensis* protein hydrolysate

| Gene | **∆∆**Ct | Ratio | Fold change | T Test |
| --- | --- | --- | --- | --- |
| **Acadm** | **5.12** | **0.03** | **-34.66** | **0.003** |
| Adipoq | — | — | — | — |
| Cpt1a | -0.05 | 1.04 | 1.04 | 0.611 |
| **Gcg** | **-1.34** | **2.53** | **2.53** | **0.439** |
| Htr2c | -0.26 | 1.20 | 1.20 | 0.016 |
| Klf9 | -0.23 | 1.18 | 1.18 | 0.012 |
| Srebf1 | 0.19 | 0.88 | -1.14 | 0.943 |
| Ucp2 | -0.29 | 1.22 | 1.22 | 0.002 |
| Adipor1 | -0.02 | 1.02 | 1.02 | 0.682 |
| Prkaa1 | -0.68 | 1.61 | 1.61 | 0.001 |
| Ppard | 0.03 | 0.98 | -1.02 | 0.840 |
| Pparg | -0.15 | 1.11 | 1.11 | 0.618 |
| Ntrk2 | -0.03 | 1.02 | 1.02 | 0.625 |
| Bdnf | 0.11 | 0.93 | -1.08 | 0.217 |
| Lpl | — | — | — | — |
| Cebpa | 0.83 | 0.56 | -1.78 | 0.021 |
| **Adra2b** | **-1.01** | **2.02** | **2.02** | **0.106** |
| Fabp4 | -0.30 | 1.23 | 1.23 | 0.840 |
| Gcgr | — | — | — | — |
| Glp1r | 0.08 | 0.94 | -1.06 | 0.900 |
| Lep | -0.26 | 1.20 | 1.20 | 0.627 |
| Ppara | 0.04 | 0.97 | -1.03 | 0.921 |
| Map3k5 | 0.05 | 0.97 | -1.03 | 0.875 |
| Slc27a1 | -0.13 | 1.10 | 1.10 | 0.293 |
| Acaca | -0.23 | 1.18 | 1.18 | 0.332 |
| Ghsr | -0.98 | 1.97 | 1.97 | 0.279 |
| Scd1 | -0.24 | 1.18 | 1.18 | 0.009 |
| Nfkb1 | -0.21 | 1.16 | 1.16 | 0.356 |
| Insr | -0.07 | 1.05 | 1.05 | 0.783 |
| Hmgcs1 | 0.02 | 0.99 | -1.01 | 0.790 |
| Apoa4 | — | — | — | — |
| Cntfr | 0.21 | 0.86 | -1.16 | 0.337 |
| Grpr | 0.41 | 0.75 | -1.33 | 0.118 |
| Hmgcs2 | -0.01 | 1.01 | 1.01 | 0.960 |
| Ptpn1 | 0.20 | 0.87 | -1.14 | 0.059 |
| Slc2a4 | -0.61 | 1.52 | 1.52 | 0.072 |
| Retn | 0.48 | 0.72 | -1.39 | 0.060 |
| Ghrl | 0.89 | 0.54 | -1.85 | 0.285 |
| Pyy | — | — | — | — |
| Adcy1 | 0.00 | 1.00 | -1.00 | 0.994 |
| Fas | -0.43 | 1.35 | 1.35 | 0.192 |
| Tnf | 0.47 | 0.72 | -1.39 | 0.608 |
| Acsl1 | -0.12 | 1.09 | 1.09 | 0.196 |
| Hmgcr | 0.40 | 0.76 | -1.31 | 0.041 |
