# Supplementary material for: Anti-obesity effects of Spirulina platensis protein hydrolysate by modulating brain-liver axis in high-fat diet fed mice
Source: PLoS One. 2019 Jun 20;14(6):e0218543. doi: 10.1371/journal.pone.0218543 (PMC6586325; doi:10.1371/journal.pone.0218543)
Supplement: S4 Table — (DOCX) [file pone.0218543.s005.docx]

S4 Table Gene changes in liver tissues of high fat diet fed-mice treated with distilled water and *Spirulina platensis* protein hydrolysate

| Gene | ∆∆Ct | Ratio | Fold change | T Test |
| --- | --- | --- | --- | --- |
| Acadm | -0.11 | 1.08 | 1.08 | 0.765 |
| Adipoq | — | — | — | — |
| **Cpt1a** | **-1.01** | **2.02** | **2.02** | **0.000** |
| Gcg | — | — | — | — |
| Htr2c | — | — | — | — |
| Klf9 | -0.51 | 1.42 | 1.42 | 0.013 |
| Srebf1 | 0.26 | 0.84 | -1.20 | 0.649 |
| Ucp2 | -0.85 | 1.80 | 1.80 | 0.004 |
| Adipor1 | -0.40 | 1.31 | 1.31 | 0.001 |
| Prkaa1 | 0.12 | 0.92 | -1.09 | 0.919 |
| **Ppard** | **2.57** | **0.17** | **-5.96** | **0.153** |
| Pparg | -0.01 | 1.01 | 1.01 | 0.952 |
| **Ntrk2** | **1.61** | **0.33** | **-3.06** | **0.394** |
| Bdnf | 0.11 | 0.93 | -1.08 | 0.742 |
| Lpl | — | — | — | — |
| Cebpa | 0.34 | 0.79 | -1.26 | 0.188 |
| Adra2b | 0.09 | 0.94 | -1.06 | 0.903 |
| **Fabp4** | **-3.96** | **15.51** | **15.51** | **0.000** |
| Gcgr | -0.10 | 1.07 | 1.07 | 0.596 |
| Glp1r | — | — | — | — |
| Lep | -0.16 | 1.12 | 1.12 | 0.733 |
| Ppara | -0.28 | 1.22 | 1.22 | 0.081 |
| Map3k5 | -0.61 | 1.53 | 1.53 | 0.048 |
| **Slc27a1** | **-2.43** | **5.37** | **5.37** | **0.018** |
| Acaca | -1.10 | 2.15 | 2.15 | 0.023 |
| Ghsr | -0.59 | 1.51 | 1.51 | 0.457 |
| **Scd1** | **-1.25** | **2.38** | **2.38** | **0.000** |
| **Nfkb1** | **-1.25** | **2.38** | **2.38** | **0.328** |
| Insr | 0.15 | 0.90 | -1.11 | 0.291 |
| **Hmgcs1** | **-1.43** | **2.70** | **2.70** | **0.000** |
| Apoa4 | -0.43 | 1.35 | 1.35 | 0.218 |
| Cntfr | — | — | — | — |
| Grpr | — | — | — | — |
| Hmgcs2 | 0.29 | 0.82 | -1.23 | 0.002 |
| Ptpn1 | -0.73 | 1.66 | 1.66 | 0.002 |
| **Slc2a4** | **-1.70** | **3.25** | **3.25** | **0.008** |
| **Retn** | **-5.29** | **38.99** | **38.99** | **0.000** |
| Ghrl | — | — | — | — |
| Pyy | — | — | — | — |
| Adcy1 | — | — | — | — |
| Fas | -0.17 | 1.12 | 1.12 | 0.499 |
| Tnf | 0.39 | 0.76 | -1.31 | 0.547 |
| Acsl1 | -0.55 | 1.46 | 1.46 | 0.003 |
| **Hmgcr** | **-1.23** | **2.34** | **2.34** | **0.000** |
