# Supplementary figures and images for: Anti-obesity effects of Spirulina platensis protein hydrolysate by modulating brain-liver axis in high-fat diet fed mice
Source: PLoS One. 2019 Jun 20;14(6):e0218543. doi: 10.1371/journal.pone.0218543 (PMC6586325; doi:10.1371/journal.pone.0218543)

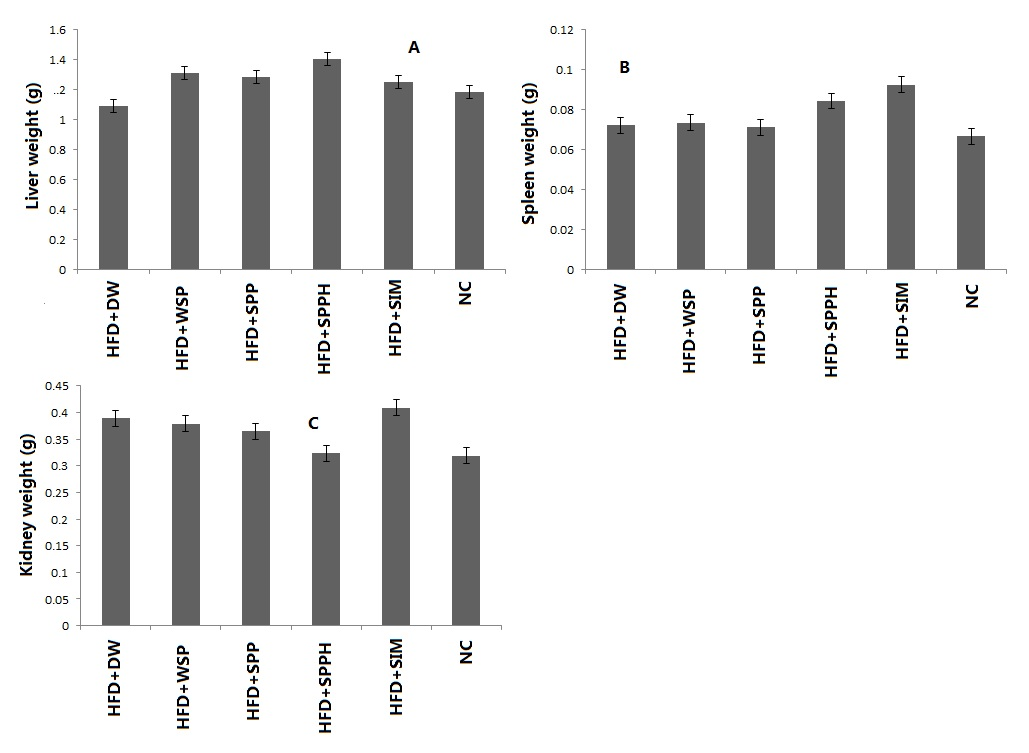


**S1 Fig** The liver, spleen and kidney coefficients under different treatments.

Supplement: S1 Fig — (DOCX) [file pone.0218543.s006.docx]
